# Divide Precisely and Proliferate Safely: Lessons From Budding Yeast

**DOI:** 10.3389/fgene.2018.00738

**Published:** 2019-01-10

**Authors:** Roberta Fraschini

**Affiliations:** Dipartimento di Biotecnologie e Bioscienze, Università degli Studi di Milano-Bicocca, Milan, Italy

**Keywords:** SPB, centrosome, mitotic spindle, genomic stability, aneuploidy

## Abstract

A faithful cell division is essential for proper cellular proliferation of all eukaryotic cells; indeed the correct segregation of the genetic material allows daughter cells to proceed into the cell cycle safely. Conversely, errors during chromosome partition generate aneuploid cells that have been associated to several human pathological conditions, including cancer. Given the importance of this issue, all the steps that lead to cell separation are finely regulated. The budding yeast *Saccharomyces cerevisiae* is a unicellular eukaryotic organism that divides asymmetrically and it is a suitable model system to study the regulation of cell division. Humans and budding yeast are distant 1 billion years of evolution, nonetheless several essential pathways, proteins, and cellular structures are conserved. Among these, the mitotic spindle is a key player in chromosome segregation and its correct morphogenesis and functioning is essential for genomic stability. In this review we will focus on molecular pathways and proteins involved in the control mitotic spindle morphogenesis and function that are conserved from yeast to humans and whose impairment is connected with the development of human diseases.

## Introduction

In the last decades budding yeast has been largely used as a model system to unravel molecular mechanisms of cell life. *Saccharomyces cerevisiae* is a unicellular eukaryotic organism, its cells have the same subcellular organization as those of multicellular organisms but they are easier to manipulate. In the budding yeast system several methodologies can be easily applied: genetic, biochemical, cytological, genomic approaches (Botstein and Fink, 1988), and high-throughput technologies. Several processes and proteins are conserved from yeast to human cells despite their distance from an evolutionary point of view and their misregulation is involved in disease development. The sequence of the genes and functional complementation studies have revealed that at least 20% of human genes known to have a role in disease have functional equivalents in yeast (Douzery et al., 2004). In addition, a systematic humanization analysis revealed that 47% of the yeast genes can be replaced by their human orthologs, indicative of conserved functions despite large sequence divergence (Kachroo et al., 2015).

The importance of basic research using budding yeast is highlight by three Nobel Prizes in Physiology or Medicine assigned to scientist for their discoveries of key proteins or processes in yeast. Professors L. Hartwell, J. Rothman, and Y. Ohsumi used classical forward budding yeast genetic approaches to reveal fundamental cell biological processes in eukaryotes; genetic analysis was conducted in parallel with microscopic analysis of the mutants and followed by biochemical and cell biology studies. Importantly, the conservation of the basic cellular processes across eukaryotes explains the great impact of these studies for understanding biology and disease.

Studies in budding yeast have revealed the essential functions of the centromeres, DNA sequences that allow the assembly of specialized multiprotein complexes, called kinetochores, that connect chromosomal DNA with mitotic spindle fibers in a bipolar way and ensure proper chromosome segregation during mitosis. A surveillance mechanism called Spindle Assembly Checkpoint (SAC) has been described in yeast and is conserved throughout evolution; the SAC delays anaphase onset in case of lack of biorientation, that is the correct binding of the two sister chromatids kinetochores to opposite spindle poles. Importantly, altered SAC function allows premature mitotic exit and can cause chromosome missegregation thus leading to aneuploid daughter cells, i. e., with an abnormal chromosome number. Interestingly, both decreased and increased SAC gene expression are found in tumors in mice (Sotillo et al., 2007; Ricke et al., 2011), in addition mutations in *MAD1*, *MAD2*, *BUB1*, *BUBR1*, and *BUB3* are found in human cancers and over expression of the same genes is associated with elevated proliferation index and metastatic potential in several solid tumors (Yuan et al., 2006; Wang et al., 2015). These data link cancer occurrence with SAC function, chromosome segregation defects and aneuploidy (for a complete overview on this topic see Simonetti et al., 2018).

The mitotic spindle is essential to allow proper partitioning of the genetic material between the daughter cells, it has a conserved structure and its malfunctions are at the basis of several human diseases, as described below. In budding yeast a bipolar spindle is formed during S phase, concomitantly with DNA duplication, while in animal cells the spindle apparatus is built during mitosis. However, in all eukaryotic cells the mitotic spindle is formed by microtubules (MTs), cylindric structures made by protofilaments of α- and β-tubulin heterodimers assembled in a head-to-tail fashion. Each MT has a dynamic fast growing end (plus end) and a slow growing end (minus end). MTs are associated with several proteins involved in regulation of spindle dynamics and with motor proteins that allow spindle positioning in the cell and intracellular transport. Before sister chromatids separation in anaphase, MTs plus ends bind the chromosomes via their kinetochores in a bipolar way thus ensuring their correct segregation in the daughter cells.

## MTOC Structure and Function

**FIGURE 1:**
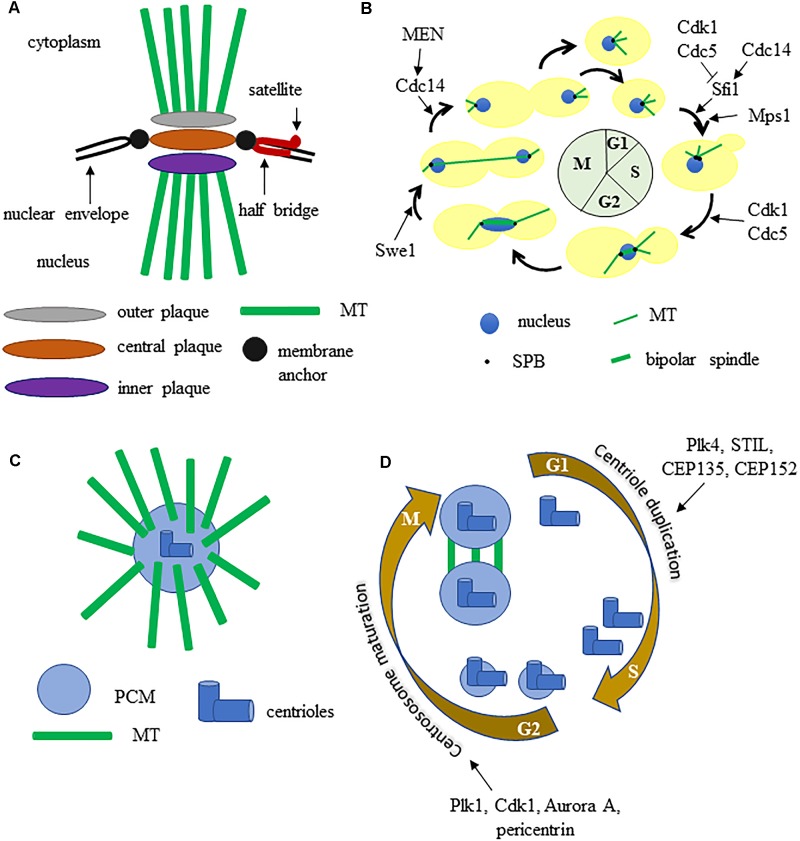
**(A)** graphic representation of the budding yeast spindle pole body. **(B)** the SPB and spindle cycle in budding yeast, some key regulators are indicated. **(C)** schematic structure of a metazoan centrosome. **(D)** centrosome duplication and maturation cycle, some key regulators are indicated. See text for details.

Eukaryotic cells contain microtubule organizing centers (MTOC) or centrosomes that allow MT nucleation in coordination with cell cycle progression. In *S. cerevisiae* the mitotic spindle is build thanks to the spindle pole body (SPB), the functional equivalent of multicellular eukaryotes centrosome. SPBs are approximately 2 megadaltons complexes embedded in the nuclear envelope and are able to nucleate both nuclear and cytoplasmic MTs, thus SPBs play a critical role in mitotic spindle formation and positioning. The SPB has a multilayered structure that consists of an outer plaque that faces the cytoplasm and emanates cytoplasmic microtubules, a central plaque, and an inner plaque that faces the nucleoplasm and emits nuclear MTs (Byers and Goetsch, 1974; Figure 1A). Attached to one side of the central plaque there is the half-bridge, essential for SPB duplication as it serves for the assembly of the satellite, the precursor of the daughter SPB. Given the crucial function of SPB, its components are encoded by essential genes. SPBs and human MTOCs share conserved proteins with common functions (Cavanaugh and Jaspersen, 2017), therefore the yeast SPB is good model to study centrosome function.

The SPB cycle is tightly connected with other cell cycle events (Figure 1B). The SPB duplicates once each cell cycle, just like chromosomes: during S phase the MTs bind the kinetochores and the SPBs separate from each other, thus allowing the formation of a short bipolar spindle. After separation, each SPB inherits a half bridge, essential for its duplication in the following cell cycle. During anaphase the SPBs further move away from each other toward the cortex of the mother and the daughter cell (Fraschini, 2017). SPB duplication is restricted once per cell cycle thanks to an oscillation between the activities of Cdk1 kinase and Cdc14 phosphatase. Sfi1 is a Centrin/Cdc31 binding protein, conserved from yeast to humans, that plays a key role in SPB cycle. During S phase and early mitosis Sfi1 is phosphorylated by Cdk1 and by Cdc5 and blocks the process of SPB duplication. After anaphase onset, the protein phosphatase Cdc14 is active and dephosphorylates Sfi1 thus allowing the maturation of the half-bridge and therefore daughter SPB formation (Elserafy et al., 2014).

Spindle pole body function is regulated by multiple proteins during the cell cycle and several SPB components have been shown to be phosphorylated *in vivo* (Keck et al., 2011). A genome-wide screen for the substrates of the cyclin dependent kinase Cdk1 identified some SPB components (Spc42, Spc29, Mps2, Bbp1, Sfi1) and suggested that Spc110, Cnm67, Kar1 may be Cdk1 substrates as well (Ubersax et al., 2003). Mps1 is a protein kinase involved in regulation of SPB duplication and phosphorylates three SPB components: Spc98, Spc110, Spc42. The polo-like kinase Cdc5 localizes to SPBs and over expression of a version of Cdc5 lacking the polo-box results in the formation of Spc42-containing structures in the cytoplasm. Pericentrin/Spc110 might be phosphorylated by Cdc5, since one of its phosphorylation sites matches the Cdc5 kinase consensus sequence and an affinity capture-western experiment showed that Cdc5 and Spc110 interact. The protein kinase Swe1 is an important cell cycle regulator, as it blocks entry into mitosis through inhibitory phosphorylation of the catalytic subunit of the cyclin-dependent kinase Cdk1 (*S. cerevisiae* Cdc28) in case of replication stress and alterations in actin cytoskeleton or cytokinetic structures (Booher et al., 1993). Interestingly Swe1, homolog of human Wee1, localizes at SPBs (Bartholomew et al., 2001), it is involved in mitotic spindle dynamics and it interacts with the outer plaque component Centrosomin/Spc72 (Raspelli et al., 2015, 2018).

Studies on SPB helped to reveal the protein composition and function of animal cells MTOC, that have a partly different structure. Each centrosome contains a pair of nine-fold symmetrical centrioles embedded in a proteinaceous matrix known as the pericentriolar material (PCM), which comprises proteins required for microtubule nucleation and cell cycle regulators (Figure 1C). Concomitantly with DNA replication, centrioles duplicate themselves to form a new centrosome then, during late G2 phase, centrosomes undergo maturation, which results in PCM expansion and recruitment of the conserved γ-tubulin rings. Polo-like kinase 1 (Plk-1) regulates the increase of PCM that accompanies mitotic entry by controlling the localization of γ-tubulin, Spd-2, and Cnn/CDK5RAP2. During prophase centrosomes separate, generate fibers and form a bipolar spindle (Nigg and Holland, 2018; Figure 1D).

## MTOC Malfunctions and Genetic Diseases

Studies in budding yeast have revealed the role of some proteins essential for SPB function and their homologs are involved in genetic diseases in human (Table 1). For example Spc110, the yeast homolog of pericentrin, is an essential component of the SPB inner plaque that stimulates binding with γ-tubulin and interacts with Cmd1, the homolog of human Calmodulin, a calcium sensor involved in the propagation of intracellular calcium signals. Missense mutations in one of the three genes coding for Calmodulin are associated with catecholaminergic polymorphic ventricular tachycardia (CPVT), early-onset severe long QT syndrome (esLQT), and idiopathic ventricular fibrillation (IVF) (Vassilakopoulou et al., 2015). Mutations that inactivate the *PCNT* gene, that encodes pericentrin, a structural component of the centrosome, can be found in all patients affected by Microcefalicosteodysplastic primordial dwarfism type II (MOPDII) (Ren et al., 2008). MOPDII is a rare and complex human autosomal recessive genetic disease, and individuals affected show primordial growth problems that are present before birth. Since pericentrin is essential for mitotic spindle organization, mitotic progression and chromosome segregation, loss of its function causes defective recruitment of several proteins to the centrosome and inability to properly assemble microtubules, thus disrupting the mitotic cycle and cell division. These severe mitotic problems cause a dramatic reduction in the number of cells of both the growing embryo and the adult organism, resulting in small head, and body size (Delaval and Doxsey, 2010).

**Table 1:** Summary of conserved genes involved in MTOC dynamics/signaling and the correlated genetic diseases.

*S. cerevisiae*	*H. sapiens*	Disease	Protein function	Localization yeast/human
*CDC5*	Plk1	Glioma, several types of carcinoma, melanoma, colorectal cancers, breast cancer, prostate cancer	MTOC separation	SPB outerplaque/centrosome
*SFI1*	hSFI1	–	MTOC duplication	SPB half bridge/centriole
*CDC31*	Centrin	–	MTOC duplication	SPB half bridge/centriole
–	hPOC5	Idiopathic scoliosis	Binds Centrin and hSFI1	–
*SPC72*	Centrosomin CDK5RAP2/Cnn	Autosomal primary recessive microcephaly (MCPH)	MTOC organization	SPB outer plaque/centrosome
–	CEP63, CEP135, CEP152, CPAP/MCPH6, STIL/MCPH7	Autosomal primary recessive microcephaly (MCPH)	Centriole duplication	Centrosome
–	ASPM, WDR62	Autosomal primary recessive microcephaly (MCPH)	Centriole duplication	Spindle poles
*SPC110*	Pericentrin	Microcephalic osteodysplastic primordial dwarfism 2 (MOPD2)	MTOC maturation	SPB central plaque/centrosome
*NUD1*	Centriolin	Stem cell myeloproliferative disorder (MPD)	MTOC signaling	SPB outer plaque/centriole
*CMD1*	Calmodulin	CPV Tachycardia, early-onset severe long QT syndrome (esLQT), idiopathic ventricular fibrillation (IVF)	Calcium binding protein (MTOC structure)	SPB central plaque/nucleus and cytoplasm
*TUB4*	GCP1	Microcephaly, cortical dysplasia	γ-tubulin	SPB outer and inner plaque/centrosome
*SPC97*	GCP2	Dilated cardiomyopathy	γ-tubulin small complex	SPB outer and inner plaque/centrosome
*SPC98*	GCP3	Dilated cardiomyopathy	γ-tubulin small complex	SPB outer and inner plaque/centrosome
*CDC15*	MST1/2	Breast cancer, soft tissue sarcoma	Signaling kinase (Hippo pathway)	SPB/spindle poles
*DBF2/DBF20*	LATs/NDR	Breast cancer, astrocytoma	Signaling kinases (Hippo pathway)	SPB/spindle poles
*MOB1*	MOB1	Colorectal and lung cancers	Co-activator (Hippo pathway)	SPB/spindle poles

The homolog of Centrosomin (Cnn) is Spc72, an essential component of the SPB outer plaque, that interacts with Nud1, the yeast counterpart of human Centriolin. The SPBs components Cdc31 and Sf1 are the yeast counterparts of Centrin and hSFI1. Autosomal primary recessive microcephaly (MCPH) is a disorder in neurogenesis caused by at least nine genes, six of which encode centrosome components (CEP63, CEP135, CEP152, CDK5RAP2/Cnn, CPAP /MCPH6, and STIL/MCPH7) and two encode proteins associated with spindle poles (ASPM and WDR62) (Gilmore and Walsh, 2013). Mutations in these genes alter the precise centriole duplication process and therefore cause deregulation of centrosome number in cells. A translocation between chromosomes 8 and 9 disrupts Centriolin function and is associated with stem cell myeloproliferative disorder (MPD) (Ren et al., 2013). Idiopathic scoliosis is a complex disease with polygenic background that leads to a spinal deformity. Human POC5 dysfunction is associated with idiopathic scoliosis (Patten et al., 2015), being hPOC5 a protein that binds centrin and that is important for centriole duplication (Azimzadeh et al., 2009) together with Centrin and hSFI1 (Martinez-Sanz and Assairi, 2016).

## Centrosome Amplification and Cancer

Centrosome amplification can cause the formation of multipolar spindle during mitosis, thus resulting in daughter cells with unbalanced genetic material. In yeast, SPB duplication is restricted by precise molecular mechanisms, as described above (Elserafy et al., 2014) and defective spindle or chromosome biorientation are sensed by the SAC, as described in the introduction (Stukenberg and Burke, 2015). The target of the checkpoint is the mitotic exit network (MEN), a pathway that governs the transition from mitosis to the G1 phase of the cell cycle (Hotz and Barral, 2014). The MEN pathway is essential for proper coordination of nuclear division and exit from mitosis. Key MEN components are localized at the SPBs, that are therefore an important signaling platforms for mitosis progression. The MEN is conserved and in metazoan it is called the Hippo pathway; importantly, recently it has been shown that the core components of the Hippo pathway cooperate with p53 to suppress tumorigenesis (Furth et al., 2018). This cooperation occurs at multiple levels, for example in response to stress LATS2 blocks MDM2, a negative p53 regulator, thus causing p53 accumulation and activation (for a complete overview on this topic see Furth et al., 2018).

In the context of tumorigenesis, centrosome abnormalities and amplification are frequently detected in a wide range of solid cancers, myeloma, lymphomas and leukemias, and have been associated with multipolar cell divisions, chromosomal instability and aneuploidy (Chan, 2011). Cells with less or more than 2 centrosomes can form anastral, monopolar or multipolar spindles that can lead to chromosome missegregation and therefore aneuploidy. Centrosome defects can be found in early-low grade lesions, and are rarely observed in normal tissue, suggesting a possible role in tumor initiation. Recent studies show that centrosome aberrant number causes tumor formation in mice (Levine et al., 2017), centrosome amplification is correlated with high-grade tumors, disease progression and poor prognosis and also it enhances the aggressive nature of already transformed cells (Godinho and Pellman, 2014). Finally, several oncogenes and tumor suppressors have been localized to the centrosomes suggesting that they might contribute to centrosome anomalies (Tang et al., 2011). Centrosome alterations trigger a p53-response that arrest the cell cycle, indeed p53-proficient cells tolerate well variations of centrosomes copy number, while cancer cells defective in p53 frequently display centrosome anomalies (Lambrus et al., 2015).

Extracentrosomes can arise basically through two mechanisms: centriole overduplication or accumulation of mature centrosomes by aborted cell division, cell fusion, or centrosome clustering. It has been shown that the majority of centrosome aberrations in the primary tumor types are due to overduplication. However, in solid tumors other types of centrosome aberrations, originated from centrosome clustering or failed cytokinesis, are also found (Cosenza et al., 2017). Spindle multipolarity is strongly correlated with anaphase bridges, which cause DNA breaks that usually block cytokinesis, thus leading to centrosome amplification.

Centrosome amplification can be due to a deregulation of its duplication cycle that is controlled by many positive and negative regulators, such as members of the Cdk, Aurora/Ipl1, Polo-like, and NIMA families of conserved cell cycle kinases (Brownlee and Rogers, 2013). Some of these kinases are likely hyperactive in tumor tissue, since several centrosomal proteins are hyperphosphorylated in breast tumor cells compared to normal breast tissue (Lingle et al., 2005). Another key player is STIL, that interacts with the Polo-like kinase 4 (Plk4): its depletion leads to a decrease in centriole numbers while its excess activity causes extra centrioles (Habedanck et al., 2005). Cdk inhibitor p27^Kip1^ levels and localization are highly regulated during the cell cycle and it acts to ensure proper centrosome amplification (Sharma et al., 2012). Recently, p27 involvement in centrosome duplication and cancer has also been studied at the systems levels (Barberis and Verbruggen, 2017), this kind of approach integrates experimental and computational data and allows the prediction of how perturbation of a protein can influence a biological process under analysis.

## Spindle Alignment and Cell Polarity

Asymmetry is very important for the life of a cell: during development it drives the cellular fate, indeed in stem cells the asymmetric division discriminates the daughter cell that will differentiate and the other cell that will maintain the ability to proliferate. The asymmetry of a cell is built thanks to the polarization of several factors. In most eukaryotic cells astral MTs emanating from the centrosomes are captured by protein complexes at the cell cortex, align the mitotic spindle to the polarity axis of the cell and drive asymmetric division of the cell (Siller and Doe, 2009). Also budding yeast divides asymmetrically: the daughter cell originates from a bud that emerges from the mother cell, and the bud is the equivalent of the stem cells that retain the possibility to divide, while the mother gets old.

In budding yeast the localization of polarity factors determines the bud emergence site. At the beginning the bud grows in a polarized way, then the growth becomes isotropical and the bud becomes round shaped. The bud neck separates the mother from the daughter cell and it is the place where cells will divide. Since the division site is defined during late G1 phase, before DNA replication and mitotic spindle formation, in order to ensure proper chromosome partitioning during mitosis, the spindle must be correctly positioned and aligned with respect to the mother-bud axis (Lee et al., 2000). Yeast is the first model for which the mechanisms for spindle positioning have been described: an actin dependent and a dynein dependent pathway guide the process. In addition, the spindle orientation checkpoint (SPOC) blocks mitotic exit and cytokinesis in case of spindle mispositioning or misorientation (Caydasi and Pereira, 2012). If the checkpoint fails, cytokinesis occurs even if the nucleus divides into the mother cell, thus causing the formation of aneuploid cells.

The existing SPB is also called old SPB, while the one originated by duplication is called new SPB. Usually, the old SPB migrates into the bud thanks to cytoplasmatic MTs that contact the bud cortex. The two SPBs undergo different steps of regulation, for example differential Kar9 recruitment drives the movement of the selected SPB to the bud neck and helps spindle alignment (Liakopoulos et al., 2003). Several pathways contribute to SPB asymmetry: Kar9 is preferentially recruited to astral MTs emanated from the old SPB and this is governed by the SPB inheritance network (SPIN) and the MEN (Lengefeld et al., 2017). Recent data revealed that the asymmetry of the SPBs is due to spatial cues rather than different maturation (Lengefeld et al., 2018). Similarly, in animal cells the old MTOC nucleates more astral MTs and is surrounded by more PCM than the new one, indicating that the new one is immature while the old one is mature and fully active (Lerit and Rusan, 2013).

Also in insect cells several data indicate that centrosome inheritance is consonant with cell fate decision. The stem cells of *Drosophila* male germline divide asymmetrically and produce a cell that differentiate and a cell that is totipotent. It has been observed that the old centrosome migrates in the cell that is able to renew while the new centrosome is inherited by the cell that is going to differentiate (Yamashita et al., 2007). Similar data were obtained in mouse radial glia progenitors and in *Drosophila* neuroblasts (Januschke et al., 2011), indicating that asymmetry of MTOCs and fate decision is a common feature of eukaryotic cells.

The centrosome plays an important role in brain development, indeed aberrant centrosome behavior is linked to inherited microcephaly. Microcephaly is the result of premature neural differentiation due to an insufficient number of symmetric division of neuroprogenitor cells before differentiation, that starts with the first asymmetric division, during cerebral cortex formation. Proper centrosome segregation ensures correct spindle orientation and the succession of several symmetric cell divisions before the beginning of differentiation (Morrison and Kimble, 2006). Several genes that encode for proteins implicated in centrosome function and spindle orientation are mutated in microcephaly in humans: MCPH5 or ASPM (abnormal spindle-like microcephaly associated), WDR62/MCPH2, and CEP63 (Thornton and Woods, 2009). However, not all forms of microcephaly are linked with spindle orientation defects, indicating that the causes of deficiencies in brain development are still partially unclear.

## Closing Remarks

The model organism *S. cerevisiae* offers powerful genetic tools to dissect the molecular pathways that control centrosome structure and number. In budding yeast the genetic analyses and manipulation are simple and fast, it is possible to synchronize cells in different cell cycle phases, to perform genetic screenings, and in addition the yeast two-hybrid assay allows detecting labile protein-protein interactions in the centrosome.

Despite structural differences, the yeast SPB carries many conserved proteins of the centriole and/or centrosome machinery in metazoans. Thus, it is becoming of growing interest to compare the structure and function of SPB with centrosomes and studies in budding yeast can elucidate the role of centrosomal proteins in physiological conditions. Altogether the knowledge provided by the studies on SPB structure and function in budding yeast will also improve our understanding of the molecular basis of important human diseases thus helping in developing new biomarkers and therapies.

## Author Contributions

RF wrote and revised the manuscript.

## Conflict of Interest Statement

The author declares that the research was conducted in the absence of any commercial or financial relationships that could be construed as a potential conflict of interest. The handling Editor and author RF declared their involvement as co-editors in the Research Topic, and confirm the absence of any other collaboration.
